# American Journal of Obstetrics and Diseases of Women and Children

**Published:** 1871-09

**Authors:** 


American Journal of Obstetrics and Diseases of Women and Children.

This very valuable quarterly is now published by Wm. Baldwin & Co., Publishers, New York. Price $5 per annum.

